# Phase I/II study of S-1 combined with weekly docetaxel in patients with metastatic gastric carcinoma

**DOI:** 10.1038/sj.bjc.6604312

**Published:** 2008-03-25

**Authors:** S R Park, H K Kim, C G Kim, I J Choi, J S Lee, J H Lee, K W Ryu, Y-W Kim, J-M Bae, N K Kim

**Affiliations:** 1 Department of Gastric Cancer, Research Institute and Hospital, National Cancer Center, 809 Madu1, Ilsan, Goyang, Gyeonggi 410-769, Republic of Korea

**Keywords:** S-1, docetaxel, metastatic gastric carcinoma, phase I/II study

## Abstract

We designed a phase I/II trial of S-1 combined with weekly docetaxel to determine the maximum tolerated dose (MTD) and recommended dose (RD) and to evaluate the efficacy and toxicity in metastatic gastric carcinoma (MGC). Patients with measurable disease received S-1 orally b.i.d. on days 1–14 and docetaxel intravenously on days 1 and 8 every 3 weeks. In phase I (*n*=30), each cohort received escalating doses of S-1 (30–45 mg m^−2^ b.i.d.) and docetaxel (25–40 mg m^−2^); MTD was 45 mg m^−2^ b.i.d. S-1/35 mg m^−2^ docetaxel and RD was 40 mg m^−2^ b.i.d. S-1/35 mg m^−2^ docetaxel. Dose-limiting toxicities included grade 3 elevated liver enzymes, gastric perforation, grade 3 diarrhoea/fatigue, febrile neutropenia with grade 3 anorexia/fatigue, and neutropenic infection with grade 3 stomatitis/anorexia. In phase II (*n*=52), the overall response rate was 66.7% (95% confidence interval (CI): 53.8–79.6%) and the median time to progression and overall survival were 6.5 months (95% CI: 4.9–8.1) and 13.7 months (95% CI: 9.9–17.5), respectively. The most common grade 3/4 toxicity was neutropenia (29.4%), and febrile neutropenia/neutropenic infection occurred in 19.6% of patients. Non-haematological toxicities were generally mild. There was one treatment-related death due to pneumonitis. S-1 combined with weekly docetaxel is active in MGC with moderate toxicities.

Despite its decreasing incidence over the past few decades, gastric cancer remains one of the major cause of death due to cancer worldwide (Parkin *et al*, 2005). Survival benefits have been demonstrated by systemic chemotherapy in patients with locally advanced or metastatic gastric carcinoma (MGC) (Pyrhonen *et al*, 1995; Glimelius *et al*, 1997); however, the results of most combination regimens have been unsatisfactory, with median survival times of 6–9 months (Webb *et al*, 1997; Vanhoefer *et al*, 2000; Ohtsu *et al*, 2003). To date, the most commonly used combination chemotherapies have been based on fluorouracil (5-FU) and/or cisplatin, with 5-FU/cisplatin (FP) and epirubicin/cisplatin/5-FU being currently regarded as reference treatments. Although a recent phase III trial showed that patients treated with docetaxel combined with FP had superior survival to patients treated with FP alone, the former regimen also had severe toxicities, thereby limiting its application (Van Cutsem *et al*, 2006). In addition, cisplatin-based chemotherapy is frequently associated with an unfavourable toxicity profile, including severe emesis, neurotoxicity, and nephrotoxicity. Continuous intravenous infusion of 5-FU also results in inconvenience to patients and catheter-related complications. Therefore, there is a need to develop active, but less-toxic, chemotherapy regimens, which include new active compounds.

S-1 is a novel oral fluoropyrimidine, consisting of tegafur, 5-chloro-2,4-dihydroxypyridine, a dihydropyrimidine dehydrogenase inhibitor, and potassium oxonate, which inhibits orotate phosphoribosyl transferase in the gastrointestinal tract, thereby suppressing gastrointestinal toxicity caused by the phosphoribosylation of 5-FU (Shirasaka *et al*, 1996). S-1 has demonstrated significant activity in advanced gastric cancer, achieving response rates of 26–49% with good safety profiles in several phase II trials (Sakata *et al*, 1998; Koizumi *et al*, 2000; Chollet *et al*, 2003).

Docetaxel, which inhibits microtubule depolymerisation, has been widely used in the treatment of MGC, with response rates of 16–24% when used as a single agent in phase II trials (Sulkes *et al*, 1994; Bang *et al*, 2002). Compared with the 3-weekly regimen, docetaxel administered once weekly has a favourable safety profile, including myelosuppression (Schuette *et al*, 2005; Bria *et al*, 2006; Camps *et al*, 2006).

Due to the activity of S-1 and docetaxel in MGC, and their synergistic activity in gastric cancer cell lines and xenografts (Takahashi *et al*, 2005; Wada *et al*, 2006), we designed a phase I/II trial of S-1 combined with weekly docetaxel in patients with MGC to determine the maximum tolerated dose (MTD) and recommended dose (RD) of these agents when used together, and to evaluate their efficacy and toxicity.

## PATIENTS AND METHODS

### Eligibility

Patients were eligible for this trial if they were over 18 years of age with histologically proven MGC, unidimensionally measurable disease, an ECOG (Eastern Cooperative Oncology Group) performance status of 0–2, and possessed adequate baseline haematological function (ANC (absolute neutrophil count) ⩾1.5 × 10^9^ l^−1^, platelet count ⩾100 × 10^9^ l^−1^), hepatic function (serum aspartate aminotransferase and alanine aminotransferase ⩽2.5 times ULN (upper limit of normal) and serum bilirubin ⩽ULN), and renal function (serum creatinine ⩽ULN). Patients in the phase II part had received no prior chemotherapy, including adjuvant chemotherapy, whereas patients in the phase I part were permitted up to two previous chemotherapy regimens, except for prior taxane or S-1, and were required to have discontinued chemotherapy for at least 4 weeks before participating in this study.

Patients were excluded if they had a history of other malignancies within the previous 3 years, had severe comorbid conditions, or lacked the ability to comply with the requirements of the protocol. Patients receiving drugs with potential interactions with S-1 (e.g., flucytosine, allopurinol, and phenytoin) were also excluded. All patients provided written informed consent, and the protocol was approved by our Institutional Review Board.

### Pretreatment evaluations

Baseline evaluations included medical history, physical examination, ECOG performance status, complete blood cell count, serum chemistries and electrolytes, urinalysis, urine pregnancy test (for women), 24-h urine creatinine clearance, chest X-ray, computed tomography, electrocardiogram, and recording of concomitant medications.

### Treatment and study design

During each 3-week cycle, patients received oral S-1 twice daily (within 1 h after morning and evening meals) on days 1–14 and a 1 h intravenous infusion of docetaxel on days 1 and 8. All the patients received oral dexamethasone (4 mg twice daily for 4 doses, starting 12 h before docetaxel) and parenteral pheniramine maleate (45.5 mg) prophylactically. Prophylactic administration of granulocyte colony-stimulating factor and antiemetics was not allowed; however, secondary prophylaxis or therapy with antiemetics in subsequent cycles was allowed. Treatment was continued in the absence of disease progression or unacceptable toxicity for a maximum of 12 cycles.

During phase I, each cohort of at least three patients was treated with escalating doses of S-1/docetaxel: 30 mg m^−2^ b.i.d./25 mg m^−2^ (level 1), 35 mg m^−2^ b.i.d./25 mg m^−2^ (level 2), 35 mg m^−2^ b.i.d./30 mg m^−2^ (level 3), 40 mg m^−2^ b.i.d./30 mg m^−2^ (level 4), 40 mg m^−2^ b.i.d./35 mg m^−2^ (level 5), 45 mg m^−2^ b.i.d./35 mg m^−2^ (level 6), and 45 mg m^−2^ b.i.d./40 mg m^−2^ (level 7). Dose escalation was continued until at least one-third of the patients in a given cohort showed dose-limiting toxicity (DLT) during the first cycle. Before escalating to the next dose level, all three patients should have received at least one treatment cycle. If none of the first three treated patients developed DLT during the first cycle at a specific dose level, dose escalation was continued. If one out of the first three treated patients developed DLT at any dose level, three additional patients were entered at the same dose level; if only one in six patients at a given level experienced a DLT, dose escalation was continued. The MTD was defined as the dose level at which one-third or more of patients experienced a DLT. The RD for the subsequent phase II study was defined as the dose level preceding the attainment of the MTD.

Dose-limiting toxicity was defined as any of the following: (1) grade 4 neutropenia lasting at least 7 days; (2) grade 3/4 neutropenia associated with infection or fever (⩾38.3°C as single temperature or ⩾38.0°C for 1 h); (3) grade 3 thrombocytopenia with grade 3/4 haemorrhage; (4) grade 4 thrombocytopenia; (5) grade 3/4 non-haematological toxicity other than alopecia or nausea/vomiting relieved by antiemetic therapy; (6) grade 3/4 nausea/vomiting not reduced to grade ⩽1 with aggressive antiemetic support; or (7) inability of the patient to take ⩾75% of the planned chemotherapy dose during the treatment period.

Phase II was performed using the RD determined during phase I.

### Dose modifications

The next chemotherapy cycle was delayed if patients had ANC <1.5 × 10^9^ l^−1^, platelet count <100 × 10^9^ l^−1^, or any grade >1 non-haematological toxicity, excluding alopecia. Docetaxel dose was reduced by 20% in subsequent cycles if patients experienced grade 3/4 neutropenia associated with infection or fever (⩾38.3°C as single temperature or ⩾38.0°C for 1 h), grade 4 thrombocytopenia, grade 3 non-haematological toxicity, grade 2/3 neurological toxicity, or recurrent fluid retention. Docetaxel treatment on day 8 was delayed to day 10 for ANC <1.5 × 10^9^ l^−1^, platelet count <75 × 10^9^ l^−1^, or grade ⩾2 non-haematological toxicity on the day of scheduled treatment. On day 10, docetaxel dose was reduced by 50% for ANC of 0.5 × 10^9^ l^−1^–1.0 × 10^9^ l^−1^ or platelet count of 50 × 10^9^ l^−1^–75 × 10^9^ l^−1^, and omitted for other toxicities. S-1 dose was reduced by 20% in subsequent cycles if patients experienced grade 3/4 neutropenia associated with infection or fever, grade 4 thrombocytopenia, a second occurrence of a grade 2 non-haematological toxicity, or any grade 3 non-haematological toxicity. If patients experienced a grade 4 non-haematological toxicity, docetaxel and S-1 were definitively interrupted or continued at doses 50% less than the starting dose.

### Evaluation during chemotherapy

During phase I, complete blood cell count and serum chemistries were monitored twice weekly and once weekly, respectively, during the first cycle, except for the first week and on days 1 and 8 of each subsequent cycle. During phase II, complete blood cell count and serum chemistries were monitored weekly during the first two cycles and on days 1 and 8 of each subsequent cycle.

### Assessment of efficacy and toxicity

Computed tomography scans were performed every two cycles to evaluate the tumour response, which was assessed according to the Response Evaluation Criteria in Solid Tumors (Therasse *et al*, 2000). Objective responses were confirmed by a second evaluation 4–6 weeks later. Time to progression (TTP) was calculated from the date of first chemotherapy cycle to the date of disease progression, and overall survival (OS) was calculated from the date of first chemotherapy cycle to either the date of death due to any cause or the date of the last follow-up visit. Toxicity was graded according to the National Cancer Institute Common Toxicity Criteria (version 2.0).

### Phase II study: statistical planning and analysis

The primary end point for the phase II part of the study was the objective response rate, and the secondary endpoints were TTP, OS, and safety. A two-stage optimal design proposed by Simon was used to determine the sample size of phase II (Simon, 1989). Assuming *P*_0_=0.3, *P*_1_=0.5, with *α*=0.05 and *β*=0.2, the first stage required at least 6 out of 15 patients to have a response before proceeding to the second stage. An additional 31 assessable patients were to be enrolled; if 19 or more out of the 46 assessable patients would have a response, the treatment would be considered sufficiently active. Assuming that 10% of patients could not be evaluated, the planned sample size of the phase II part was 52 patients. The parameters TTP and OS were estimated using the Kaplan–Meier method.

## RESULTS

### Phase I

#### Patient characteristics

A total of 30 patients, of median age 50 years (range: 22–71) entered phase I from September 2004 to September 2005. Six patients entered at dose level 1, three each at dose levels 2–4, six each at dose levels 5 and 6, and three at dose level 7. Patient characteristics are summarised in Table 1. Most patients had an ECOG performance status of 1 (93.3%) and multiple metastases involving two or more organ systems (86.7%). All patients had metastatic disease; three had recurrent disease and had received prior adjuvant chemotherapy (5-FU plus mitomycin C). Another patient had received adjuvant doxifluridine and radiotherapy for resected pancreatic cancer 5 years earlier.

#### Dose-limiting toxicity, maximum tolerated dose, and recommended dose

Chemotherapy toxicities per patient including DLTs during the first cycle are summarised in Table 2. At dose level 1 (30 mg m^−2^ b.i.d. S-1 plus 25 mg m^−2^ docetaxel), one of the first three patients experienced a DLT (grade 3 aspartate aminotransferase elevation) but none of the three additional patients experienced a DLT. During dose escalation, DLT did not develop until dose level 7 (45 mg m^−2^ b.i.d. S-1 plus 40 mg m^−2^ docetaxel), at which two out of three patients experienced DLTs (grade 3 febrile neutropenia and grade 3 infection with neutropenia with grade 3 stomatitis/anorexia). Therefore, the next three patients were entered at dose level 6 (45 mg m^−2^ b.i.d. S-1 plus 35 mg m^−2^ docetaxel), but two of these patients (two out of six) had DLTs (grade 3 diarrhoea with grade 3 fatigue and febrile neutropenia with grade 3 anorexia/fatigue). An additional three patients were entered at dose level 5 (40 mg m^−2^ b.i.d. S-1 plus 35 mg m^−2^ docetaxel), but one (one out of six) experienced a DLT (gastric perforation at the tumour site on the first day of the first cycle). Therefore, level 6 was considered as the MTD and level 5 was defined as the RD for the ensuing phase II study.

### Phase II

#### Patient characteristics

From October 2005 to July 2006, 52 patients were enrolled in the phase II study: 38 (73.1%) males and 14 (26.9%) females of median age 53 years (range: 23–70). Most of the patients (94.2%) had an ECOG performance status of 1, and 45 (86.5%) had multiple metastases involving two or more organ systems. Metastatic sites included the abdominal lymph nodes (94.2%), peritoneum (50.0%), liver (34.6%), and others (25.0%) (Table 1). All patients had metastatic disease, with three having recurrent disease after prior subtotal gastrectomy.

#### Efficacy

**Figure 1:**
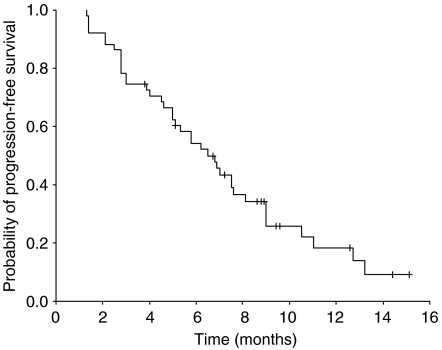
Time to progression for all evaluable patients in phase II (*n*=51).

**Figure 2:**
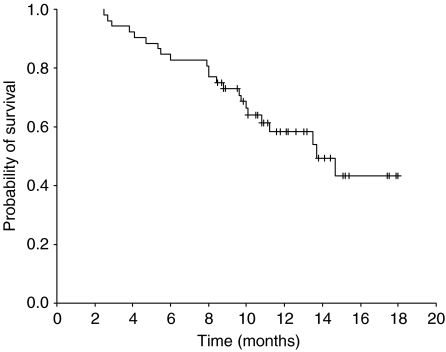
Overall survival for all eligible patients in phase II (*n*=52).

Out of the 52 patients, 51 could be evaluated for response, whereas 1 patient was lost to follow-up after day 1 of the first chemotherapy cycle. Two patients (3.9%) achieved complete response and 32 (62.7%) had partial response, making the overall response rate 66.7% (95% confidence interval (CI): 53.8–79.6%). Twelve patients (23.5%) had stable disease and five (9.8%) had progressive disease. All objective responses were confirmed by follow-up computed tomography at least 4 weeks after the initial documentation of response. The median duration of response was 6.3 months (range: 1.6 to 13.8+). The median follow-up time was 13.1 months (range: 8.5–18.0), during which the median TTP was 6.5 months (95% CI: 4.9–8.1 months) (Figure 1) and the median OS was 13.7 months (95% CI: 9.9–17.5) (Figure 2). The one-year survival rate was 58.5% (95% CI: 44.2–72.8%).

#### Treatment delivered

A total of 385 cycles were administered, with a median of 8 per patient (range: 1–12). Treatment was delayed for a median of 6 days (range: 4–28) in 70 cycles (18.2%) in 43 patients (82.7%), mainly because of grade 2–4 neutropenia (11 cycles), infection without neutropenia (10 cycles), grade 2 stomatitis (7 cycles), febrile neutropenia/infection with neutropenia (5 cycles), and abnormal liver function test (5 cycles). Seventeen cycles were delayed due to reasons unrelated to disease or treatment, including pending imaging studies to evaluate response or at the patient's request. Dose reduction of S-1 was required in 148 (38.4%) cycles in 27 patients (51.9%), primarily due to febrile neutropenia/infection with neutropenia (51 cycles), grade 3 or recurrent grade 2 stomatitis (36 cycles), grade 3 or recurrent grade 2 fatigue (14 cycles), grade 3 or recurrent grade 2 diarrhoea (12 cycles), grade 3 infection without neutropenia (9 cycles), and recurrent grade 2 abdominal pain (7 cycles). Dose reduction of docetaxel was required in 100 cycles (26.0%) in 18 patients (34.6%), primarily due to febrile neutropenia/infection with neutropenia (51 cycles), grade 3 fatigue (13 cycles), grade 3 infection without neutropenia (9 cycles), grade 3 stomatitis (7 cycles), and grade 3 diarrhoea (7 cycles). The relative dose intensities of S-1 and docetaxel were 85.6% (319.5 mg m^−2^ per week) and 91.8% (21.4 mg m^−2^ per week), respectively.

The reasons for discontinuation of treatment were disease progression (*n*=32, 61.5%), adverse events (*n*=3, 5.8%) (one patient each with grade 4 docetaxel-induced pneumonitis, grade 4 pneumonia, and grade 3 peripheral neuropathy), loss to follow-up (*n*=3, 5.8%), and patient refusal (*n*=3, 5.8%). The remaining 11 patients finished the planned maximum of 12 cycles of chemotherapy; 8 of these patients are currently being followed without chemotherapy, whereas 3 had disease progression while off chemotherapy.

#### Toxicity

Fifty-one patients were assessable for toxicity. Table 3 summarises chemotherapy toxicities per patient. The most common grade 3/4 haematological toxicities were neutropenia (29.4% of patients) and leukopenia (29.4%). Grade 3/4 febrile neutropenia and grade 3 infection with neutropenia each occurred in five patients (9.8%). All these patients were successfully treated with antibiotics and granulocyte colony-stimulating factor. Grade 3 anaemia occurred in three patients (5.9%), but no patient experienced grade 3/4 thrombocytopenia.

Non-haematological toxicities were generally mild-to-moderate and manageable. Grade 4 non-haematological toxicity occurred in only one patient (2.0%) who experienced docetaxel-induced pneumonitis and died of this disease. The most common grade 3 non-haematological toxicity was infection without neutropenia (*n*=8, 15.7%): pneumonia (*n*=4), periungal infection (*n*=2), cellulitis (*n*=1), and urinary tract infection (*n*=1). Other grade 3 non-haematological toxicities were grade 3 fatigue (9.8% of patients), stomatitis (5.9%), constipation (5.9%), diarrhoea (3.9%), aspartate aminotransferase/alanine aminotransferase elevation (3.9%), anorexia (2.0%), nausea (2.0%), peripheral neuropathy (2.0%), hand-foot syndrome (2.0%), and docetaxel-induced pneumonitis (2.0%).

#### Second-line chemotherapy

During the follow-up period, 36 (69.2%) out of the 52 patients received second-line chemotherapy: 17 received irinotecan/cisplatin, 8 received oxaliplatin/5-FU/leucovorin, 3 received irinotecan/5-FU/leucovorin, 3 received capecitabine/cisplatin, and 5 received other regimens. Out of these 36 patients, 34 were evaluable for efficacy, 9 (26.5%) achieved a partial response, 10 (29.4%) had stable disease, and 15 (44.1%) showed progression. The median TTP of second-line chemotherapy was 2.8 months (95% CI: 2.0–3.6).

## DISCUSSION

We have shown here that S-1 combined with weekly docetaxel is a highly active first-line chemotherapy regimen for MGC. The overall response rate of 66.7%, median TTP of 6.5 months, and median OS of 13.7 months are comparable to results of trials using older (Webb *et al*, 1997; Vanhoefer *et al*, 2000; Ohtsu *et al*, 2003) or newly developed chemotherapeutic agents (Roth *et al*, 2000; Ridwelski *et al*, 2001; Al-Batran *et al*, 2004; Bouche *et al*, 2004; Park *et al*, 2004; Chun *et al*, 2005; Kim *et al*, 2005; Moehler *et al*, 2005; Giordano *et al*, 2006; Kang *et al*, 2006; Orditura *et al*, 2006; Van Cutsem *et al*, 2006), including oxaliplatin, irinotecan, capecitabine, and other docetaxel-containing regimens. With the latter agents, response rates ranged from 9 to 60%, median TTP from 1.9 to 6.9 months, and median OS from 5.7 to 12.0 months (Roth *et al*, 2000; Ridwelski *et al*, 2001; Al-Batran *et al*, 2004; Bouche *et al*, 2004; Park *et al*, 2004; Chun *et al*, 2005; Kim *et al*, 2005; Moehler *et al*, 2005; Giordano *et al*, 2006; Kang *et al*, 2006; Orditura *et al*, 2006; Van Cutsem *et al*, 2006).

Recently, phase III trials of S-1 alone and/or S-1 combined with cisplatin showed that S-1 alone had non-inferior OS compared with infusional 5-FU (11.4 *vs* 10.8 months, non-inferiority *P*<0.001), and then S-1 combined with cisplatin had superior OS to S-1 alone (13.0 *vs* 11.0 months, *P*=0.0366) (Boku *et al*, 2007; Narahara *et al*, 2007). Considering the potential of the S-1-based combination regimen, the combination of S-1 and the new cytotoxic agent in the present study deserves comparison in further phase III trials.

During phase I of our trial, we determined that the RD and treatment schedule for phase II was 40 mg m^−2^ b.i.d. S-1 on days 1–14 and 35 mg m^−2^ docetaxel on days 1 and 8 of each 3-week cycle. Two recent trials of S-1 combined with docetaxel used different doses and schedules; the first used 40 mg m^−2^ S-1 b.i.d. on days 1–14 and 40 mg m^−2^ docetaxel on day 1 of each 3-week cycle (Yoshida *et al*, 2006), and the second used 40 mg m^−2^ b.i.d. S-1 on days 1–14 and 40 mg m^−2^ docetaxel on day 1 of each 4-week cycle (Yamaguchi *et al*, 2006). Despite these variations, the DLTs in phase I of these trials included neutropenia and complicated neutropenia, similar to our trial (Yoshida *et al*, 2004; Yamaguchi *et al*, 2006). Although a direct comparison between other phase II trials and our trial is difficult, one previous trial, which had the same planned dose intensity of S-1 and a different dose intensity of docetaxel as our trial, showed similar efficacy, with an overall response rate of 56.3% (compared with 66.7% in our trial), a median TTP of 7.3 months (compared with 6.5 months), and a median OS of 14.3 months (compared with 13.7) (Yoshida *et al*, 2006). About 10% of patients in this trial, however, had locally advanced disease, whereas all of our patients had metastatic disease (Yoshida *et al*, 2006). Moreover, the patients in this trial received a median of four cycles (in a 3-week cycle) and had a median response duration of only 5.1 months (Yoshida *et al*, 2006). This discrepancy between the relatively low number of median treatment cycles per patient and long median TTP may not be usual treatment outcomes. In our study, patients received a median eight cycles of chemotherapy and had a median response duration of 6.3 months. Another previous trial, which used a lower dose intensity (40 mg m^−2^ b.i.d. S-1 on days 1–14 and 40 mg m^−2^ docetaxel on day 1 of each 4-week cycle) compared with our trial, showed inferior efficacy, with an overall response rate of 46% and a median progression-free survival of 4.1 months (Yamaguchi *et al*, 2006). It may be attributed to the weekly regimen that a higher dose of docetaxel could be determined as RD in our trial than in previous two trials; docetaxel administered once weekly has lower toxicity with comparable efficacy compared with the 3-weekly regimen (Schuette *et al*, 2005; Bria *et al*, 2006; Camps *et al*, 2006).

The regimen used in the present trial resulted in a generally low incidence of grade 3/4 haematological toxicities, including neutropenia (29.4%), and non-haematological toxicities. Febrile neutropenia and infection with neutropenia, however, occurred in a relatively high proportion of patients (19.6%). This may be related to the relatively high incidence of grade 1/2 stomatitis (80.4%) and diarrhoea (72.5%), in that disruption of the mucosal barrier may make patients susceptible to infection. This high incidence of grade 1/2 non-haematological toxicities may be associated with the long treatment duration, a median of eight cycles per patient. Due to the frequency of febrile neutropenia or infection with neutropenia and the actual dose intensity of S-1 and docetaxel in phase II of our trial, we propose that the RD of this regimen be lowered to 35 mg m^−2^ b.i.d. S-1 on days 1–14 and/or 30 mg m^−2^ docetaxel on days 1 and 8 of each 3-week cycle, or prophylactic granulocyte colony-stimulating factor be used.

Notably, this regimen resulted in a low incidence of nausea and vomiting despite the absence of primary prophylactic antiemetics. Grade 2 nausea and vomiting occurred in 31.4 and 17.6% of patients, respectively, and grade 3 nausea developed in only one patient (2.0%). During the entire treatment period, only 19 patients (36.5%) in 49 cycles (12.7%) were given a serotonin antagonist for secondary prophylaxis during subsequent cycles or therapy. The current regimen compares favourably with cisplatin, irinotecan, oxaliplatin, or infusional 5-FU-containing regimens, in which the incidence of grade 3/4 nausea/vomiting ranged from 4.9 to 26% with prophylactic antiemetics (Webb *et al*, 1997; Roth *et al*, 2000; Vanhoefer *et al*, 2000; Ridwelski *et al*, 2001; Ohtsu *et al*, 2003; Al-Batran *et al*, 2004; Bouche *et al*, 2004; Moehler *et al*, 2005; Kang *et al*, 2006; Van Cutsem *et al*, 2006).

This regimen also resulted in a very low incidence of grade 2/3 hand-foot syndrome (5.9%), which is quite troublesome and has been reported to occur in 12.9% to more than 50% of patients treated with capecitabine, another oral fluoropyrimidine, combined with docetaxel (Park *et al*, 2004; Chun *et al*, 2005; Kim *et al*, 2005; Giordano *et al*, 2006).

Taken together, we conclude that S-1 combined with weekly docetaxel is a highly active outpatient regimen in MGC with moderate toxicity. Further studies with appropriate dose modifications are warranted.

## Figures and Tables

**Table 1:** Patient characteristics

**Characteristic**	**Phase I**	**Phase II**
*No. of patients*	30	52
*Median age in years (range)*	50 (27–71)	53 (23–70)
		
*Gender*		
Male	20 (66.7%)	38 (73.1%)
Female	10 (33.3%)	14 (26.9%)
		
*ECOG performance status*		
0	2 (6.7%)	3 (5.8%)
1	28 (93.3%)	49 (94.2%)
		
*Metastatic organ site*		
Abdominal lymph node	25 (83.3%)	49 (94.2%)
Peritoneum	19 (63.3%)	26 (50.0%)
Liver	8 (26.7%)	18 (34.6%)
Others	14 (46.7%)	13 (25.0%)
		
*No. of metastatic organ sites*		
1	4 (13.3%)	7 (13.5%)
2	17 (56.7%)	23 (44.2%)
⩾3	9 (30.0%)	22 (42.3%)
		
*Prior treatment*		
Total gastrectomy	1 (3.3%)	0 (0%)
Subtotal gastrectomy	2 (6.7%)	3 (5.8%)
Adjuvant 5-FU+MMC	3 (10.0%)	0 (0%)
Adjuvant doxifluridine+RT	1 (3.3%)	0 (0%)

ECOG=Eastern Cooperative Oncology Group; 5-FU=5-fluorouracil; MMC=mitomycin C; RT=radiotherapy.

**Table 2:** Toxicities at various dose levels of S-1 and docetaxel during the first cycle of phase I

	**Dose^a^ of S-1 and docetaxel**													
	**30/25 (*n*=6)**		**35/25 (*n*=3)**		**35/30 (*n*=3)**		**40/30 (*n*=3)**		**40/35 (*n*=6)**		**45/35 (*n*=6)**		**45/40 (*n*=3)**	
**Toxicity (NCI-CTC)**	**G1/2**	**G3/4**	**G1/2**	**G3/4**	**G1/2**	**G3/4**	**G1/2**	**G3/4**	**G1/2**	**G3/4**	**G1/2**	**G3/4**	**G1/2**	**G3/4**
Anaemia	4	1	3	0	2	0	2	0	4	0	6	0	3	0
Leukopenia	2	0	1	0	2	0	2	0	3	0	3	1	1	2
Neutropenia	2	0	0	1	0	1	2	0	2	0	2	2	0	3
Thrombocytopenia	0	0	0	0	0	0	0	0	0	0	0	0	1	0
Febrile neutropenia/neutropenic infection	—	0	—	0	—	0	—	0	—	0	—	1	—	2
Diarrhoea	2	0	1	0	1	0	1	0	4	0	2	1	3	0
Stomatitis	2	0	0	0	1	0	2	0	2	0	4	0	2	1
Nausea^b^	3	0	3	0	1	0	1	0	3	0	3	0	2	0
Vomiting^b^	1	0	0	0	0	0	1	0	0	0	1	0	1	0
Anorexia	3	0	2	0	3	0	2	0	5	0	5	1	2	1
Fatigue	1	0	1	0	1	0	3	0	4	0	4	2	3	0
Elevated AST/ALT	2	1	0	0	1	0	0	0	2	0	0	0	0	0
Gastric perforation	0	0	0	0	0	0	0	0	0	1	0	0	0	0

ALT=alanine aminotransferase; AST=aspartate aminotransferase; NCI-CTC=National Cancer Institute Common Toxicity Criteria.

a One dose of S-1 and docetaxel; for example, 30/25 means 30 mg m^−2^ S-1 twice on days 1–14 and 25 mg m^−2^ docetaxel on days 1 and 8.

b Prophylactic administration of antiemetics was not allowed during the first cycle.

**Table 3:** Toxicity of chemotherapy in phase II (*n*=51)

	**No. of patients (%)**			
**Toxicity (NCI-CTC)**	**Grade 1**	**Grade 2**	**Grade 3**	**Grade 4**
*Haematological*				
Leukopenia	14 (27.5)	7 (13.7)	12 (23.5)	3 (5.9)
Neutropenia	13 (25.5)	8 (15.7)	9 (17.6)	6 (11.8)
Febrile neutropenia/ infection with neutropenia	—	—	9 (17.6)	1 (2.0)
Anaemia	22 (43.1)	25 (49.0)	3 (5.9)	0 (0)
Thrombocytopenia	2 (3.9)	0 (0)	0 (0)	0 (0)
				
*Non-haematological*				
Stomatitis	20 (39.2)	21 (41.2)	3 (5.9)	0 (0)
Anorexia	24 (47.1)	24 (47.1)	1 (2.0)	0 (0)
Nausea	20 (39.2)	16 (31.4)	1 (2.0)	—
Vomiting	16 (31.4)	9 (17.6)	0 (0)	0 (0)
Diarrhoea	25 (49.0)	12 (23.5)	2 (3.9)	0 (0)
Constipation	14 (27.5)	1 (2.0)	3 (5.9)	0 (0)
Fatigue	24 (47.1)	19 (37.3)	5 (9.8)	0 (0)
Tearing	19 (37.3)	22 (43.1)	0 (0)	—
Alopecia	21 (41.2)	30 (58.8)	—	—
Oedema	38 (74.5)	6 (11.8)	0 (0)	0 (0)
Skin rash	12 (23.5)	5 (9.8)	0 (0)	0 (0)
Nail changes	16 (31.4)	22 (43.1)	—	—
Hand-foot syndrome	5 (9.8)	2 (3.9)	1 (2.0)	—
Pneumonitis	0 (0)	0 (0)	1 (2.0)	1 (2.0)
Peripheral neuropathy	34 (66.7)	3 (5.9)	1 (2.0)	0 (0)
AST/ALT elevation	15 (29.4)	5 (9.8)	2 (3.9)	0 (0)
Hyperbilirubinemia	6 (11.8)	4 (7.8)	0 (0)	0 (0)
Infection without neutropenia	0 (0)	11 (21.6)	8 (15.7)	0 (0)

ALT=alanine aminotransferase; AST=aspartate aminotransferase; NCI-CTC=National Cancer Institute Common Toxicity Criteria.

## References

[bib1] Al-Batran SE, Atmaca A, Hegewisch-Becker S, Jaeger D, Hahnfeld S, Rummel MJ, Seipelt G, Rost A, Orth J, Knuth A, Jaeger E (2004) Phase II trial of biweekly infusional fluorouracil, folinic acid, and oxaliplatin in patients with advanced gastric cancer. J Clin Oncol 22: 658–663 14966088 10.1200/JCO.2004.07.042

[bib2] Bang YJ, Kang WK, Kang YK, Kim HC, Jacques C, Zuber E, Daglish B, Boudraa Y, Kim WS, Heo DS, Kim NK (2002) Docetaxel 75 mg/m(2) is active and well tolerated in patients with metastatic or recurrent gastric cancer: a phase II trial. Jpn J Clin Oncol 32: 248–254 12324575 10.1093/jjco/hyf057

[bib3] Boku N, Yamamoto S, Shirao K, Doi T, Sawaki A, Koizumi W, Saito H, Yamaguchi K, Kimura A, Ohtsu A, Group GOSGJCO (2007) Randomized phase III study of 5-fluorouracil (5-FU) alone *vs* combination of irinotecan and cisplatin (CP) *vs* S-1 alone in advanced gastric cancer (JCOG9912). Proc Am Soc Clin Oncol 25: 200s (abstract LBA4513)

[bib4] Bouche O, Raoul JL, Bonnetain F, Giovannini M, Etienne PL, Lledo G, Arsene D, Paitel JF, Guerin-Meyer V, Mitry E, Buecher B, Kaminsky MC, Seitz JF, Rougier P, Bedenne L, Milan C (2004) Randomized multicenter phase II trial of a biweekly regimen of fluorouracil and leucovorin (LV5FU2), LV5FU2 plus cisplatin, or LV5FU2 plus irinotecan in patients with previously untreated metastatic gastric cancer: a Federation Francophone de Cancerologie Digestive Group Study––FFCD 9803. J Clin Oncol 22: 4319–4328 15514373 10.1200/JCO.2004.01.140

[bib5] Bria E, Cuppone F, Ciccarese M, Nistico C, Facciolo F, Milella M, Izzo F, Terzoli E, Cognetti F, Giannarelli D (2006) Weekly docetaxel as second line chemotherapy for advanced non-small-cell lung cancer: meta-analysis of randomized trials. Cancer Treat Rev 32: 583–587 16919884 10.1016/j.ctrv.2006.07.003

[bib6] Camps C, Massuti B, Jimenez A, Maestu I, Gomez RG, Isla D, Gonzalez JL, Almenar D, Blasco A, Rosell R, Carrato A, Vinolas N, Batista N, Giron CG, Galan A, Lopez M, Blanco R, Provencio M, Diz P, Felip E (2006) Randomized phase III study of 3-weekly *vs* weekly docetaxel in pretreated advanced non-small-cell lung cancer: a Spanish Lung Cancer Group trial. Ann Oncol 17: 467–472 16371411 10.1093/annonc/mdj115

[bib7] Chollet P, Schoffski P, Weigang-Kohler K, Schellens JH, Cure H, Pavlidis N, Grunwald V, De Boer R, Wanders J, Fumoleau P (2003) Phase II trial with S-1 in chemotherapy-naive patients with gastric cancer. A trial performed by the EORTC Early Clinical Studies Group (ECSG). Eur J Cancer 39: 1264–1270 12763215 10.1016/s0959-8049(03)00237-5

[bib8] Chun JH, Kim HK, Lee JS, Choi JY, Hwangbo B, Lee HG, Park SR, Choi IJ, Kim CG, Ryu KW, Kim YW, Bae JM (2005) Weekly docetaxel in combination with capecitabine in patients with metastatic gastric cancer. Am J Clin Oncol 28: 188–194 15803015 10.1097/01.coc.0000143877.53314.9c

[bib9] Giordano KF, Jatoi A, Stella PJ, Foster N, Tschetter LK, Alberts SR, Dakhil SR, Mailliard JA, Flynn PJ, Nikcevich DA (2006) Docetaxel and capecitabine in patients with metastatic adenocarcinoma of the stomach and gastroesophageal junction: a phase II study from the North Central Cancer Treatment Group. Ann Oncol 17: 652–656 16497828 10.1093/annonc/mdl005

[bib10] Glimelius B, Ekstrom K, Hoffman K, Graf W, Sjoden PO, Haglund U, Svensson C, Enander LK, Linne T, Sellstrom H, Heuman R (1997) Randomized comparison between chemotherapy plus best supportive care with best supportive care in advanced gastric cancer. Ann Oncol 8: 163–168 10.1023/a:1008243606668 9093725

[bib11] Kang Y, Kang WK, Shin DB, Chen J, Xiong J, Wang J, Lichinitser M, Salas MP, Suarez T, Santamaria J (2006) Randomized phase III trial of capecitabine/cisplatin (XP) *vs* continuous infusion of 5-FU/cisplatin (FP) as first-line therapy in patients (pts) with advanced gastric cancer (AGC): efficacy and safety results. Proc Am Soc Clin Oncol 24: 183s (abstract LBA4018)

[bib12] Kim JG, Sohn SK, Kim DH, Baek JH, Sung WJ, Park JY, Kim TB, Jung HY, Yu W, Lee KB (2005) Phase II study of docetaxel and capecitabine in patients with metastatic or recurrent gastric cancer. Oncology 68: 190–195 16006756 10.1159/000086773

[bib13] Koizumi W, Kurihara M, Nakano S, Hasegawa K (2000) Phase II study of S-1, a novel oral derivative of 5-fluorouracil, in advanced gastric cancer. For the S-1 Cooperative Gastric Cancer Study Group. Oncology 58: 191–197 10765119 10.1159/000012099

[bib14] Moehler M, Eimermacher A, Siebler J, Hohler T, Wein A, Menges M, Flieger D, Junginger T, Geer T, Gracien E, Galle PR, Heike M (2005) Randomised phase II evaluation of irinotecan plus high-dose 5-fluorouracil and leucovorin (ILF) *vs* 5-fluorouracil, leucovorin, and etoposide (ELF) in untreated metastatic gastric cancer. Br J Cancer 92: 2122–2128 15942629 10.1038/sj.bjc.6602649 PMC2361806

[bib15] Narahara H, Koizumi W, Hara T, Takagane A, Akiya T, Takagi M, Miyashita K, Nishizaki T, Kobayashi O, TS-1 Advanced Gastric Cancer (AGC) Clinical Trial Group (2007) Randomized phase III study of S-1 alone *vs* S-1+cisplatin in the treatment for advanced gastric cancer (The SPIRITS trial) SPIRITS: S-1 plus cisplatin *vs* S-1 in RCT in the treatment for stomach cancer. Proc Am Soc Clin Oncol 25: 201s (abstract 4514)

[bib16] Ohtsu A, Shimada Y, Shirao K, Boku N, Hyodo I, Saito H, Yamamichi N, Miyata Y, Ikeda N, Yamamoto S, Fukuda H, Yoshida S (2003) Randomized phase III trial of fluorouracil alone *vs* fluorouracil plus cisplatin *vs* uracil and tegafur plus mitomycin in patients with unresectable, advanced gastric cancer: The Japan Clinical Oncology Group Study (JCOG9205). J Clin Oncol 21: 54–59 12506170 10.1200/JCO.2003.04.130

[bib17] Orditura M, Martinelli E, Galizia G, Carlomagno C, Aurilio G, Vecchione L, Lieto E, De Placido S, Catalano G, Ciardiello F, De Vita F (2006) Weekly docetaxel and capecitabine is not effective in the treatment of advanced gastric cancer: a phase II study. Ann Oncol 17: 1529–1532 16873436 10.1093/annonc/mdl168

[bib18] Park YH, Ryoo BY, Choi SJ, Kim HT (2004) A phase II study of capecitabine and docetaxel combination chemotherapy in patients with advanced gastric cancer. Br J Cancer 90: 1329–1333 15054450 10.1038/sj.bjc.6601724 PMC2409690

[bib19] Parkin DM, Bray F, Ferlay J, Pisani P (2005) Global cancer statistics, 2002. CA Cancer J Clin 55: 74–108 15761078 10.3322/canjclin.55.2.74

[bib20] Pyrhonen S, Kuitunen T, Nyandoto P, Kouri M (1995) Randomised comparison of fluorouracil, epidoxorubicin and methotrexate (FEMTX) plus supportive care with supportive care alone in patients with non-resectable gastric cancer. Br J Cancer 71: 587–591 7533517 10.1038/bjc.1995.114 PMC2033628

[bib21] Ridwelski K, Gebauer T, Fahlke J, Kroning H, Kettner E, Meyer F, Eichelmann K, Lippert H (2001) Combination chemotherapy with docetaxel and cisplatin for locally advanced and metastatic gastric cancer. Ann Oncol 12: 47–51 11249048 10.1023/a:1008328501128

[bib22] Roth AD, Maibach R, Martinelli G, Fazio N, Aapro MS, Pagani O, Morant R, Borner MM, Herrmann R, Honegger H, Cavalli F, Alberto P, Castiglione M, Goldhirsch A (2000) Docetaxel (Taxotere)-cisplatin (TC): an effective drug combination in gastric carcinoma. Swiss Group for Clinical Cancer Research (SAKK), and the European Institute of Oncology (EIO). Ann Oncol 11: 301–306 10811496 10.1023/a:1008342013224

[bib23] Sakata Y, Ohtsu A, Horikoshi N, Sugimachi K, Mitachi Y, Taguchi T (1998) Late phase II study of novel oral fluoropyrimidine anticancer drug S-1 (1 Mtegafur-0.4 M gimestat-1 M otastat potassium) in advanced gastric cancer patients. Eur J Cancer 34: 1715–1720 9893658 10.1016/s0959-8049(98)00211-1

[bib24] Schuette W, Nagel S, Blankenburg T, Lautenschlaeger C, Hans K, Schmidt EW, Dittrich I, Schweisfurth H, von Weikersthal LF, Raghavachar A, Reissig A, Serke M (2005) Phase III study of second-line chemotherapy for advanced non-small-cell lung cancer with weekly compared with 3-weekly docetaxel. J Clin Oncol 23: 8389–8395 16293869 10.1200/JCO.2005.02.3739

[bib25] Shirasaka T, Nakano K, Takechi T, Satake H, Uchida J, Fujioka A, Saito H, Okabe H, Oyama K, Takeda S, Unemi N, Fukushima M (1996) Antitumor activity of 1 M tegafur-0.4 M 5-chloro-2,4-dihydroxypyridine-1 M potassium oxonate (S-1) against human colon carcinoma orthotopically implanted into nude rats. Cancer Res 56: 2602–2606 8653704

[bib26] Simon R (1989) Optimal two-stage designs for phase II clinical trials. Control Clin Trials 10: 1–10 2702835 10.1016/0197-2456(89)90015-9

[bib27] Sulkes A, Smyth J, Sessa C, Dirix LY, Vermorken JB, Kaye S, Wanders J, Franklin H, LeBail N, Verweij J (1994) Docetaxel (Taxotere) in advanced gastric cancer: results of a phase II clinical trial. EORTC Early Clinical Trials Group. Br J Cancer 70: 380–383 7914428 10.1038/bjc.1994.310 PMC2033505

[bib28] Takahashi I, Emi Y, Kakeji Y, Uchida J, Fukushima M, Maehara Y (2005) Increased antitumor activity in combined treatment TS-1 and docetaxel. A preclinical study using gastric cancer xenografts. Oncology 68: 130–137 16006750 10.1159/000086767

[bib29] Therasse P, Arbuck SG, Eisenhauer EA, Wanders J, Kaplan RS, Rubinstein L, Verweij J, Van Glabbeke M, van Oosterom AT, Christian MC, Gwyther SG (2000) New guidelines to evaluate the response to treatment in solid tumors. European Organization for Research and Treatment of Cancer, National Cancer Institute of the United States, National Cancer Institute of Canada. J Natl Cancer Inst 92: 205–216 10655437 10.1093/jnci/92.3.205

[bib30] Van Cutsem E, Moiseyenko VM, Tjulandin S, Majlis A, Constenla M, Boni C, Rodrigues A, Fodor M, Chao Y, Voznyi E, Risse ML, Ajani JA (2006) Phase III study of docetaxel and cisplatin plus fluorouracil compared with cisplatin and fluorouracil as first-line therapy for advanced gastric cancer: a report of the V325 Study Group. J Clin Oncol 24: 4991–4997 17075117 10.1200/JCO.2006.06.8429

[bib31] Vanhoefer U, Rougier P, Wilke H, Ducreux MP, Lacave AJ, Van Cutsem E, Planker M, Santos JG, Piedbois P, Paillot B, Bodenstein H, Schmoll HJ, Bleiberg H, Nordlinger B, Couvreur ML, Baron B, Wils JA (2000) Final results of a randomized phase III trial of sequential high-dose methotrexate, fluorouracil, and doxorubicin *vs* etoposide, leucovorin, and fluorouracil *vs* infusional fluorouracil and cisplatin in advanced gastric cancer: a trial of the European Organization for Research and Treatment of Cancer Gastrointestinal Tract Cancer Cooperative Group. J Clin Oncol 18: 2648–2657 10894863 10.1200/JCO.2000.18.14.2648

[bib32] Wada Y, Yoshida K, Suzuki T, Mizuiri H, Konishi K, Ukon K, Tanabe K, Sakata Y, Fukushima M (2006) Synergistic effects of docetaxel and S-1 by modulating the expression of metabolic enzymes of 5-fluorouracil in human gastric cancer cell lines. Int J Cancer 119: 783–791 16557585 10.1002/ijc.21879

[bib33] Webb A, Cunningham D, Scarffe JH, Harper P, Norman A, Joffe JK, Hughes M, Mansi J, Findlay M, Hill A, Oates J, Nicolson M, Hickish T, O’Brien M, Iveson T, Watson M, Underhill C, Wardley A, Meehan M (1997) Randomized trial comparing epirubicin, cisplatin, and fluorouracil *vs* fluorouracil, doxorubicin, and methotrexate in advanced esophagogastric cancer. J Clin Oncol 15: 261–267 8996151 10.1200/JCO.1997.15.1.261

[bib34] Yamaguchi K, Shimamura T, Hyodo I, Koizumi W, Doi T, Narahara H, Komatsu Y, Kato T, Saitoh S, Akiya T, Munakata M, Miyata Y, Maeda Y, Takiuchi H, Nakano S, Esaki T, Kinjo F, Sakata Y (2006) Phase I/II study of docetaxel and S-1 in patients with advanced gastric cancer. Br J Cancer 94: 1803–1808 16773074 10.1038/sj.bjc.6603196 PMC2361339

[bib35] Yoshida K, Hirabayashi N, Takiyama W, Ninomiya M, Takakura N, Sakamoto J, Nishiyama M, Toge T (2004) Phase I study of combination therapy with S-1 and docetaxel (TXT) for advanced or recurrent gastric cancer. Anticancer Res 24: 1843–1851 15274365

[bib36] Yoshida K, Ninomiya M, Takakura N, Hirabayashi N, Takiyama W, Sato Y, Todo S, Terashima M, Gotoh M, Sakamoto J, Nishiyama M (2006) Phase II study of docetaxel and S-1 combination therapy for advanced or recurrent gastric cancer. Clin Cancer Res 12: 3402–3407 16740764 10.1158/1078-0432.CCR-05-2425

